# Supplementary material for: Shared Treg dysregulation underlies psoriasis vulgaris and atopic dermatitis through the PHF19-PRC2 epigenetic axis
Source: Front Immunol. 2026 Aug 20;17:1932788. doi: 10.3389/fimmu.2026.1932788 (PMC13538097; doi:10.3389/fimmu.2026.1932788)

MR leave−one−out sensitivity analysis for LPIN2 on Psoriasis vulgaris

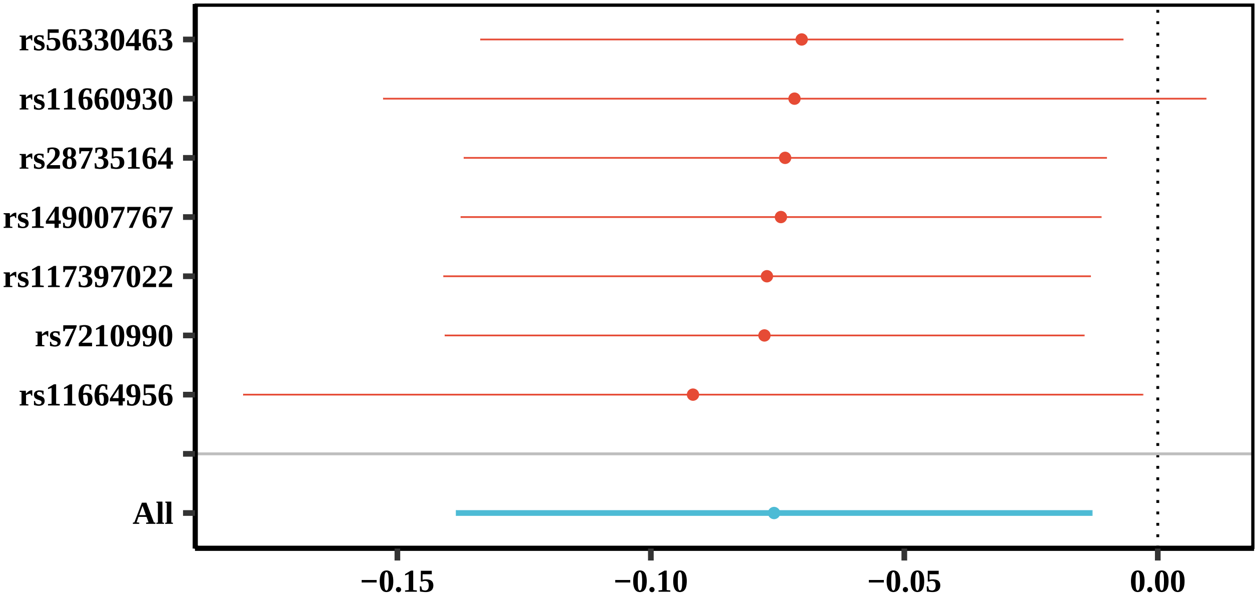

Supplement: Supplementary file 3 [file DataSheet3.zip › Additional files/Additional file 4/13.LPIN2.leaveoneout.pdf]
